# A Long-Lasting PARP1-Activation Mediates Signal-Induced Gene Expression

**DOI:** 10.3390/cells11091576

**Published:** 2022-05-07

**Authors:** Malka Cohen-Armon

**Affiliations:** The Sackler School of Medicine, Deptartment of Physiology and Pharmacology, Sagol School of Neuroscience, Tel-Aviv University, Tel-Aviv 69978, Israel; marmon@tauex.tau.ac.il

**Keywords:** PARP1 activation, H1 polyADP-ribosylation, signal transduction pathways, immediate early gene expression, histone acetylation, DNA methylation

## Abstract

This overview presents recent evidence for a long-lasting PARP1 activation by a variety of signal transduction mechanisms, mediating signal-induced gene expression and chromatin remodeling. This mode of PARP1 activation has been reported in a variety of cell types, under physiological conditions. In this mechanism, PARP1 is not transiently activated by binding to DNA breaks. Moreover, damaged DNA interfered with this long-lasting PARP1 activation.

## 1. Introduction

PolyADP-ribosylation is an evolutionary conserved, reversible post-translational modification of proteins. Numerous nuclear proteins act as substrates of the abundant nuclear polyADP-ribose polymerase 1 (PARP1) [1,2,3,4,5]. In this modification, negatively charged ADP-ribose chains constructed on chromatin-bound proteins, cause their repulsion from the negatively charged DNA [1,2,3,4,5]. In accordance, polyADP-ribosylation is a post-translational modification of proteins that causes relaxation of the highly condensed structure of the chromatin [6,7,8,9]. Histone H1, which is bound to the linker DNA, located between the nucleosomes, is a prominent substrate of PARP1. Recent findings identified eviction of polyADP-ribosylated H1 from the linker DNA in response to a variety of stimulations, resulting in DNA relaxation [8,9,10,11,12,13].

Chromatin relaxation due to polyADP-ribosylation is implicated in DNA repair. The abundant polyADP-ribose polymerase 1 (PARP1) is a major player in the initiation of DNA repair [1,2,3,4,5]. PARP1 is rapidly polyADP-ribosylated by binding to DNA breaks. PARP1 polyADP-ribosylation induces chromatin relaxation near the breaks, and the constructed ADP-ribose polymers are implicated in the recruitment of DNA-repairing enzymes to DNA breaks [1,2,3,4,5].

However, recent reports disclosed that PARP1 is also activated in the absence of DNA damage. In one mechanism, PARP1 was activated downstream to intracellular signal transduction mechanisms in a variety of cell types [10,14,15,16]. The binding of growth factors, transmitters, or hormones to their receptors in the cell membrane, as well as membrane depolarization in excitable cells, activated PARP1 via networks of intracellular signal transduction pathways, causing chromatin relaxation by polyADP-ribosylation of PARP1 and its substrate linker histone H1 [10,14,15,16]. A long-lasting polyADP-ribosylation of PARP1 was measured downstream to the MAP kinase cascade activation, and PARP1 polyADP-ribosylation mediated the phosphorylation of transcription factors and the acetylation of core histones promoting Erk-induced gene expression [10,14,15,16,17,18]. A long-lasting polyADP-ribosylation of PARP1 is also implicated in the reassembly of nucleosomes following chromatin relaxation [19], as well as in the effect of PARP1 polyADP-ribosylation on DNA methylation affecting chromatin remodeling and gene expression [20]. This review summarizes current information on this mode of PARP1 activation.

## 2. PARP1 Activation by Ca^2+^ Released from Intracellular IP_3_-Gated Stores

In a variety of cell types treated with hormones, growth factors, neurotransmitters, and membrane depolarization in excitable cells, G-protein-coupled receptors and receptor tyrosine kinases are activated [21,22]. Their activation initiates the activation of a variety of second messengers in networks of signal transduction pathways involving protein kinases and phosphatases [21,22]. In one of these signaling mechanisms, the multi-targeted phospholipase-C cleaves phosphatidyl inositols that are embedded in the cell membrane, including phosphatidylinositol 4,5-bisphosphate (PIP_2_), which is cleaved into diacylglycerol (DAG) and inositol 1,4,5-trisphosphate (IP_3_) [21,22,23]. DAG remains embedded in the membrane and acts as an anchoring molecule, and IP_3_ is released into the cytoplasm [21,22,23].

The binding of IP_3_ to specific receptors in Ca^2+^ gated stores in the endoplasmic reticulum induces intracellular Ca^2+^ release [23,24,25,26]. IP_3_-induced Ca^2+^ release from its stores in the endoplasmic reticulum, which includes the perinuclear membrane, activates a variety of Ca^2+^-dependent enzymes, including kinases and phosphatases participating in a variety of signal transduction pathways [24,25,26,27]. PARP1 was activated in response to Ca^2+^ release from the perinuclear stores into the nucleoplasm [27,28]. Ca-induced PARP1 polyADP-ribosylation was measured in isolated nuclei of a variety of cell types [27,28]. The effect of Ca^2+^ on PARP1 polyADP-ribosylation resembled the well-documented co-factor activity of Mg^2+^ ions in in vitro polyADP-ribosylation of PARP1 [28]. Divalent ions frequently act as co-factors in enzymatic reactions [29].

**Figure 1:**
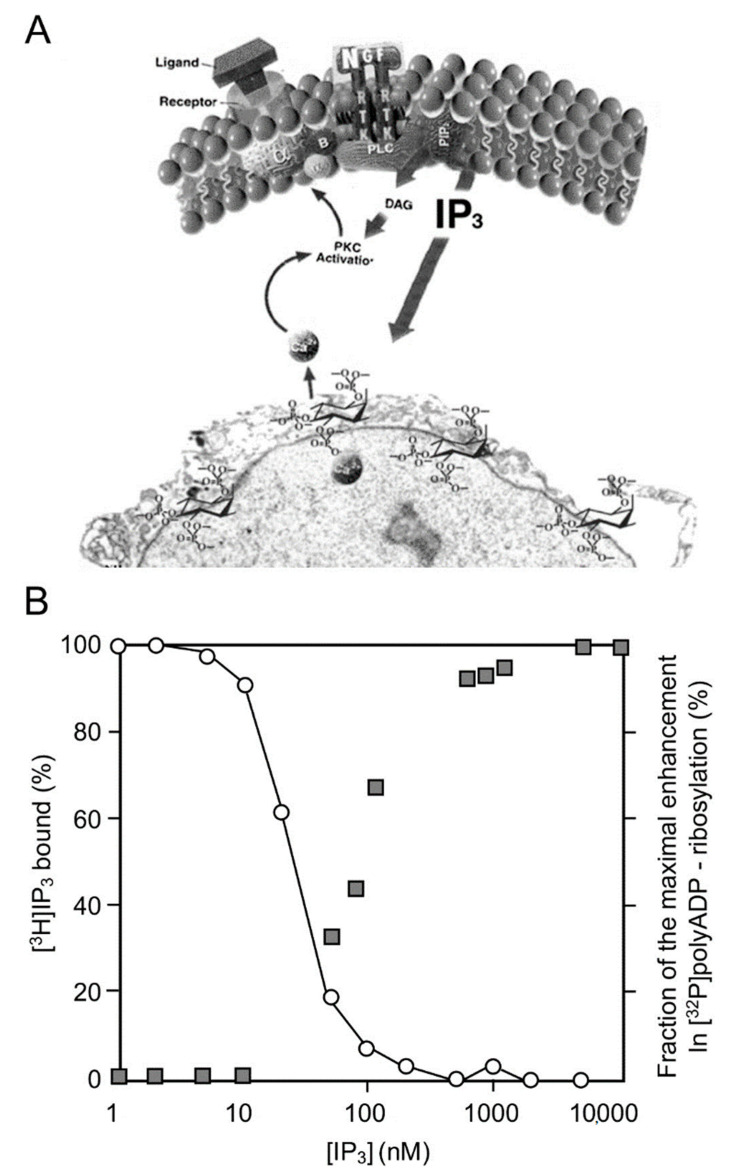
PARP1 activation by Ca^2+^ release from IP_3_-gated stores: (**A**) a schematic presentation of IP_3_ production by the cleavage of a phosphatidylinositol (PIP_2_) in the cell membrane, to DAG (diacylglycerol) and IP_3_ (Inositol triphosphate). IP_3_ induces Ca^2+^ release from IP_3_-gated Ca^2+^ stores in the perinuclear membrane of isolated nucleus (an electro-micrograph); (**B**) IP_3_-induced Ca^2+^ release activates PARP1. Right ordinate: a dose–response curve of IP_3_-induced [^32^P]polyADP-ribosylation of PARP1 in isolated nuclei of rat cerebral neurons, as measured by autography of [^32^P]labeled polyADP-ribosylated PARP1 immunoblots. Left ordinate: dose-dependent displacement of [^3^H]IP_3_ from specific IP_3_ receptors in the perinuclear membrane of isolated nuclei by unlabeled IP_3_ [28].

The IP_3_-induced release of Ca^2+^ into the nucleoplasm enhanced the polyADP-ribosylation of PARP1 in nuclei of rat cerebral neurons, as well as in nuclei of neuronal cells of the marine slug *Aplysia* [27] (Figure 1).

PARP1 was dose-dependently and instantaneously activated by IP_3_-induced Ca^2+^ -release into the nucleoplasm [28]. In addition, PARP1 was activated downstream to Ca-dependent activation of kinases, including activation of PKC and the calmodulin-dependent CAM kinases that are implicated in gene expression [30,31,32].

PKC is activated by numerous signal transduction mechanisms in a variety of cell types, including malignant cells [32,33,34]. Downstream, PKC phosphorylates RAF kinases that phosphorylate MEK kinases. MEK phosphorylation initiates the activation of the MAP kinase phosphorylation cascade, which is implicated in numerous signal transduction pathways in the cell [32,33,34].

## 3. A Long-Lasting PARP1 Activation by Phosphorylated Erk

Signals inducing phosphorylation of MEK dimers, which are bound to dimers of Erk (extracellular signal-regulated kinase) in the cytoplasm, release the phosphorylated Erk [34,35,36,37]. The released phosphorylated Erk, which lacks the nuclear localization signal (NLS), is translocated into the nucleus [35,36,37,38]. Phosphorylated Erk has a long-lasting activity in the nucleus, despite lacking the NLS signal [37,38]. A variety of transcription factors promoting the expression of numerous genes are activated by Erk-induced phosphorylation [37,39], including those promoting immediate-early genes (IEG) expression [39,40].

**Figure 2:**
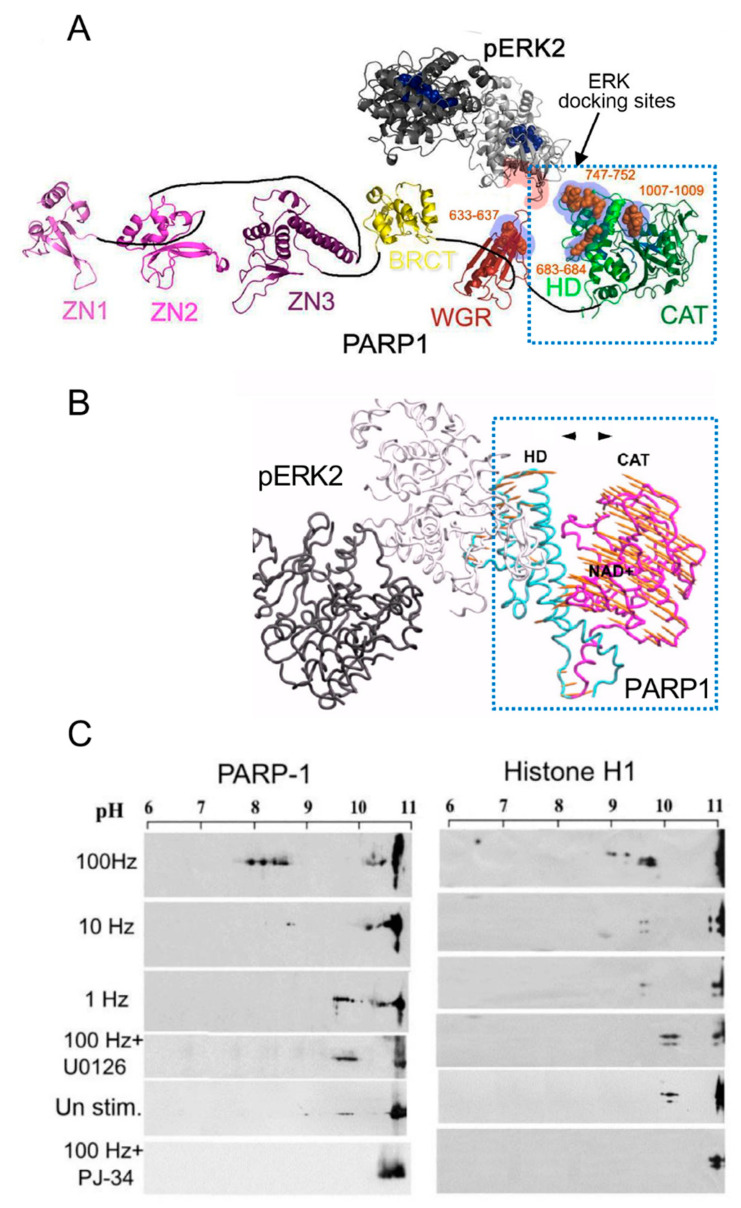
PARP1 is activated by binding to phosphorylated Erk: (**A**) a Ribbon structural model of the open conformation of PARP1 with optional consensus docking sites for phosphorylated Erk. Erk2 monomers in a homodimer (formed after Erk2 phosphorylation, PDB 2ERK) are indicated by dark and light gray ribbons. The potential Erk-binding motifs in the HD, WGR, and the CAT domain of PARP1 are indicated by orange spheres. The CRS/CD protein-binding region in Erk2 and the optional Erk-binding motifs in PARP1 are highlighted by red and blue shadows, respectively; (**B**) a model based on the calculated intra-molecular movements in PARP1 bound to homodimer of phosphorylated Erk2, predicting that the helical (HD) and the catalytic (CAT) domains of PARP1 move to opposite directions (yellow arrows), thereby exposing the NAD binding site in the CAT domain of PARP1; (**C**) polyADP-ribosylation of PARP1 and histone H1 in electrically stimulated rat cerebral neurons in primary culture. PolyADP-ribosylation of the proteins in the stimulated cerebral neurons was measured by the shift in their isoelectric points on 2D gels, which is prevented in neurons treated with either PARP or MEK inhibitors (PJ34 or U0126, respectively) [10].

Phosphorylated Erk1/2 are apparently translocated into the nucleus as homodimers [35,36] by specific transporters [35,36,37,38]. In view of the similarities between the protein binding sites of Erk1 and Erk2, as well as the major role of Erk2 activity in mammals [41], the effect of phosphorylated Erk2 on PARP1 activity has been explored. Co-immunoprecipitation of phosphorylated Erk2 with PARP1 or polyADP-ribosylated PARP1 was measured in a variety of cell types [10,15,42]. Furthermore, consensus docking sites of phosphorylated Erk were identified in PARP1 [10]. They include four sites that partially match the known docking motifs of phosphorylated Erk, found in its substrates 633KYPKK637, 683KK684, 747KKPPLL752, and 1007FNF. They are located in the WGR domain, helical domain (HD), and catalytic (CAT) domain of PARP1 (aa556–1014) [10,43,44,45,46]. In addition, a negatively charged protein-binding domain in Erk (CRS/CD region) was involved in its binding to the docking sites in PARP1 [10,15] (Figure 2). Phosphorylated Erk was co-immunoprecipitated with PARP1 in nuclear extracts of a variety of stimulated cell types, and MEK inhibitors interfered with their binding [10,15,16]. Furthermore, in a cell-free system, recombinant PARP1 was activated and highly polyADP-ribosylated upon binding of recombinant phosphorylated Erk2 [10,15]. In accordance, PARP1 activation measured in a variety of stimulated cell types was dependent on the phosphorylation of MEK [10,14,15,16,42] (Figure 2C). In addition, a long-lasting activity of phosphorylated Erk in the nuclei of these cells was dependent on PARP1 [10,15,16,17,18]. A variety of transcription factors, promoters, and modulators in the chromatin, which are prominent substrates of phosphorylated Erk, were hardly phosphorylated in PARP1 KO cells [10,18], indicating the PARP1-dependent activity of phosphorylated Erk in the chromatin. PolyADP-ribosylated PARP1 apparently acts as a platform for phosphorylated Erk, enabling phosphorylation of Erk substrates while causing local chromatin relaxation.

Notably, in a cell-free system, recombinant PARP1 was activated and polyADP-ribosylated by a recombinant phosphorylated Erk that lacks its kinase activity [15], indicating that the activation of Erk-bound PARP1 does not involve PARP1 phosphorylation [10,15]. Recombinant PARP1 bound to phosphorylated Erk2 was apparently activated due to intra-molecular modifications in PARP1, exposing the NAD^+^ binding site in its catalytic domain [10,15,46] (Figure 2A,B). Accordingly, the calculated affinity for NAD^+^ of recombinant PARP1 bound to recombinant phosphorylated Erk2 was about 70 times higher than the affinity of DNA-bound recombinant PARP1 [15]. In addition, both PARP1 and polyADP-ribosylated PARP1 equally bound phosphorylated Erk2 [10,15], outlining a mechanism that keeps PARP1 activated as long as PARP1 is bound to phosphorylated Erk [10,15,18].

The binding of PARP1 to phosphorylated Erk did not interfere with PARP1 binding to its substrate histone H1 [10,14,15,16]. Thus, the accessibility of PARP1-bound phosphorylated Erk to its substrates in the chromatin could be attributed to a local chromatin relaxation due to histone H1 polyADP-ribosylation by activated PARP1-bound to phosphorylated Erk [10,14,15]. This assumption was further supported by documented eviction of polyADP-ribosylated histone H1 from its sites in the linker DNA in response to depolarization of cerebral neurons or in response to receptors activation in the cell membrane [9,12,13,16]. Accordingly, Erk-dependent immediate-early gene expression was extensively downregulated in neurons of PARP1-KO mice, or after PARP1 silencing [10,15,18,42]. Additionally, a relatively long-lasting activity of phosphorylated Erk2 was documented in the nucleus of normal cells, compared with that period in cells prepared from PARP1 KO mice [18]. The long-lasting activity of phosphorylated Erk in the nucleus is one of the unresolved phenomena in cell biology [37,38,40]. The long-lasting binding of PARP1 to phosphorylated Erk presents a possible mechanism explaining this unresolved phenomenon [10,14,15,18,38].

**Figure 3:**
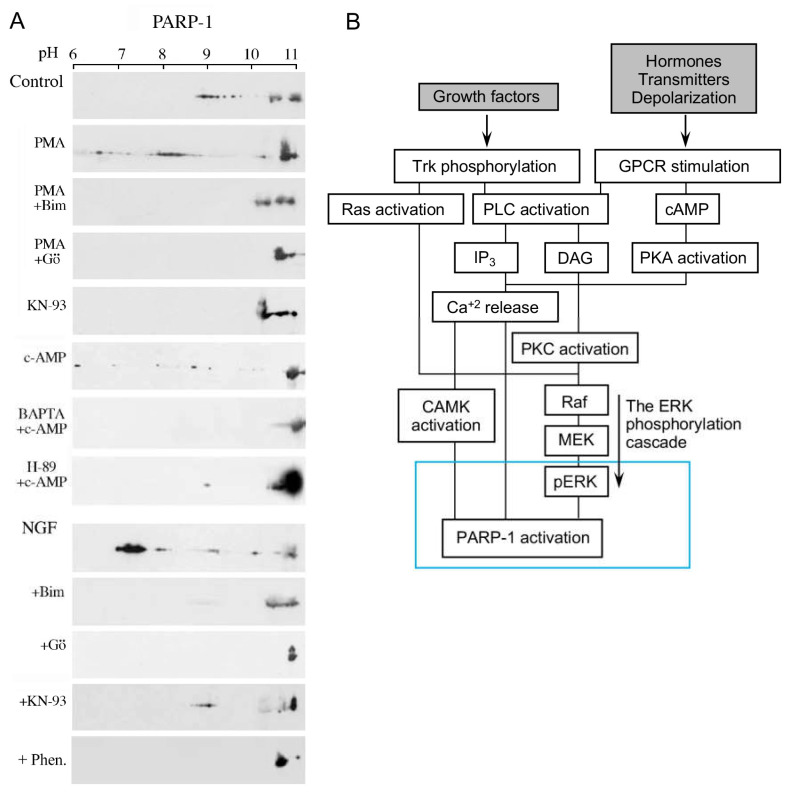
Activation and polyADP-ribosylation of PARP1 via signal-transduction Mechanisms: (**A**) PARP1 activation by signal transduction mechanisms activating G-protein-coupled receptors and receptor tyrosine kinase (TrkA), PKC, CAMKII, and PKA. The polyADP-ribosylation of PARP1 was measured by the shift in its isoelectric point on 2D gels, which is prevented by PARP inhibition (6(5H)-phenanthridinone (Phen)). The polyADP-ribosylation of PARP1 was induced in neurons treated with the indicated nerve growth factor (NGF), PKC activator (phorbol ester PMA), and PKA activator (cAMP). PARP1-induced polyADP-ribosylation was inhibited by treatments with the inhibitors of the indicated kinase PKC inhibitors, Go6796 and Bim-1, CAMK inhibitor KN-93, calcium chelator BAPTA, and PKA inhibitor H-89; (**B**) a flowchart presentation of signal transduction mechanisms mediating PARP1 activation in response to extracellular stimulations [16,17].

**Figure 4:**
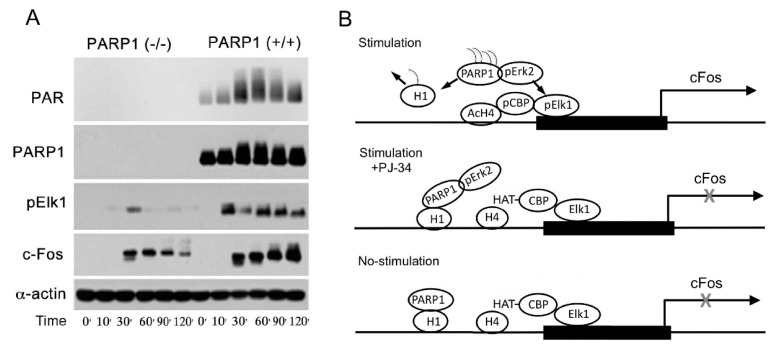
Elk phosphorylation and *cfos* expression depend on the expression of PARP1: (**A**) the presented immunoblots indicate time-dependent changes in the levels of PARP1, PAR, phosphorylated Elk1, and the protein c-Fos, following treatment of mouse embryonic fibroblasts (MEF) and PARP1-KO MEF with the PKC activator PMA; (**B**) a schematic description of PARP1-dependent expression of *cfos*. Binding of phosphorylated Erk to PARP1 activates PARP1 and polyADP-ribosylates PARP1 and linker histone H1. PolyADP-ribosylation of H1, resulting in its removal from the linker DNA, causes chromatin relaxation that facilitates the phosphorylation of transcription factor Elk1 by phosphorylated Erk bound to polyADP-ribosylated PARP1. Elk1 phosphorylation induces CBP/p300 phosphorylation that evokes their HAT activity. The resulting core histone H4 acetylation promotes *c-fos* expression [10,18].

The expression of immediate-early genes was dependent on activities of both phosphorylated Erk2 and PARP1 [10,15,16,18] and was accompanied by the eviction of polyADP-ribosylated histone H1 rendering transcription factors accessible to phosphorylation by PARP1-bound phosphorylated Erk2 [10,12,14,15]. Thus, when PARP1 is activated by a variety of signal transduction mechanisms, activating the MAP kinase phosphorylation cascade in response to extracellular signals, the synergism between PARP1 and phosphorylated Erk mediates Erk-induced gene expression (Figure 2C, Figure 3 and Figure 4A).

However, according to the structural models of PARP1-bound to DNA (PBD 4DQY), [10,46] (Figure 2A,B), DNA breaks are assumed to prevent the binding of PARP1 to phosphorylated Erk since the binding sites of phosphorylated Erk in PARP1 bound to DNA are occluded [10,15]. Accordingly, Erk-induced PARP1 polyADP-ribosylation is not anticipated in the presence of damaged DNA [10,15] and downregulation of Erk-induced gene expression is anticipated [10]. These assumptions were supported by several findings.

Ex vivo measured synaptic long-term potentiation that is mediated by Erk phosphorylation and Erk-induced immediate-early gene expression was not developed in cerebral neurons in the presence of damaged DNA, as well as in the hippocampus of PARP1 KO mice [10]. These results are in accordance with the deteriorated long-term memory of PARP1 KO mice and with long-term memory erasure by PARP1 inhibitors applied to *Aplysia*, mice, and rats before learning tests [10,47,48,49].

Downregulation of immediate-early gene expression observed in cerebral neurons of aged mice [49] was attributed to damaged DNA in these neurons. Mammalian cerebral neurons are not replaced during their life span, and the DNA of these neurons is constantly damaged by reactive oxygen species (Ros), produced in their mitochondria due to the high energy demands of cerebral neurons [50,51,52]. Accordingly, accumulation of breaks in the DNA was documented in aged cerebral neurons [50,51,52]. Thus, downregulation of Erk-induced immediate-early gene expression, which is implicated in synaptic plasticity, could be attributed to the accumulation of breaks in the DNA of these aged neurons, preventing PARP1–Erk binding and their mutual activity in the chromatin [10,15,50]. This hypothesis was further tested in cultured cerebral neurons under stress conditions inducing DNA breaks and in the presence of PARG [10]. PARG poly(ADP-ribose) glycohydrolase cleaves the ADP-ribose polymers in polyADP-ribosylated PARP1 [53]. Since polyADP-ribosylated PARP1 hardly binds to DNA, PARG activity enables the recurrent binding of PARP1 to the DNA [1,2,3].

Immediate-early gene expression implicated in synaptic plasticity was impaired in cerebral neurons treated with agents causing DNA breaks [10]. Pretreatment with PARG inhibitors restored the binding of PARP1 to phosphorylated Erk and restored Erk-induced immediate-early gene expression [10]. In accordance with these results, PARG inhibitors rescued learning deterioration in aged mice [52,54]. These findings present the difference between the activity of PARP1-bound to phosphorylated Erk and the activity of PARP1 binding to breaks in the DNA.

## 4. PARP1 PolyADP-Ribosylation-Mediating Histone Acetylation and IEG Expression

A variety of transcription factors are prominent substrates of phosphorylated Erk, either directly or via the Ca-dependent phosphorylation of the Erk substrate Rsk (ribosomal S6 kinase) [32,33]. Erk is phosphorylated downstream to numerous signal transduction mechanisms in a variety of cell types [32,33,39], and the phosphorylation of transcription factors by phosphorylated Erk promotes the expression of many genes, including immediate-early genes, [32,33,39]. The phosphorylation of transcription factors Elk1 and CREB, which are prominent substrates of phosphorylated Erk, resulted in the phosphorylation of CPB (CREB binding protein) and p300 [15,18,39]. Phosphorylation evokes their histone acetyltransferase (HAT) activity [55,56,57], and the acetylation of adjacent core histones. Core histone acetylation in their N-terminals causes a local chromatin relaxation and facilitates the initiation of DNA transcription [55,56,57]. The binding of DNA segments to acetylated histones in response to a variety of stimulations can be identified by co-immunoprecipitation using chromatin immune-precipitation (ChIP) assays [10,42]. Acetylated core histone H4 was co-immunoprecipitated with DNA segments of immediate-early genes, *cfos*, *zif268*, and *Arc* in response to membrane depolarization by electrical stimulation of cultured rodent cerebral neurons [10]. The acetylation of core histone H4 promoted the expression of immediate-early genes in response to various stimulations of cerebral neurons, and deacetyltransferases (DACT) downregulated their expression [58,59,60,61,62,63,64,65]. In view of the role of phosphorylated Erk2 in immediate-early gene expression that is implicated in synaptic plasticity [56,57,61], the interrelations between Erk phosphorylation, PARP1 activation, and core histone acetylation were tested in isolated nuclei of cerebral neurons [15]. Recombinant phosphorylated ERK2 (r-pERK2) and a recombinant mutant of phosphorylated Erk that lacks its kinase activity (KA-pERK2) were inserted into isolated nuclei of rat cerebral neurons [15]. The inserted recombinant phosphorylated Erk2 kinase caused polyADP-ribosylation of PARP1, as well as Elk1 phosphorylation and acetylation of histone H4 [15]. However, when the inserted r-pERK2 was replaced by the inactive recombinant of phosphorylated Erk2 (KA-pERK2), PARP1 was still polyADP-ribosylated, but Elk1 was not phosphorylated, and acetylated H4 was barely detectable [16]. These results clearly indicated that Elk1 phosphorylation by phosphorylated Erk2 was required for acetylation of histone H4, and the kinase activity of Erk2 was not required for the activation and polyADP-ribosylation of PARP1 [10,15]. In addition, pERK-induced Elk1 phosphorylation and H4 acetylation were both suppressed when polyADP-ribosylation was inhibited or PARP1 expression was reduced by siRNA [10,15], indicating that both polyADP-ribosylation of PARP1 and the kinase activity of Erk2 were required for Elk1 phosphorylation and H4 acetylation [10,15].

The acetylation of core histone H4 in cerebral neurons exposed to nerve growth factors was similarly mediated by PARP1 polyADP-ribosylation [10,12,15], and this mechanism was identified in other stimulated cell types [14,18]. The acetylation of histones H3 and H4 was implicated in immediate-early gene expression in stimulated cardiomyocytes [42]. Similar to the effect of PARP1 activation in cerebral neurons, the acetylation of core histones H3 and H4 was suppressed after PARP1 silencing with siRNA or inhibition [42].

In cerebral neurons, acetylation of core histone H4 mediated the expression of immediate-early genes that are implicated in synaptic plasticity and memory acquisition [47,48,62,63,64]. Numerous findings implicated core histones acetylation in long-term memory formation [59,60,65]. The HAT activity of CREB-binding protein CPB has been proven necessary for learning in a variety of animal models [58,59,60]. Inhibition of the HAT activities of CBP and P300 have been implicated in the induction of memory loss in mouse models, and inhibition of histone deacetylases restored their memory [59,66].

In ex vivo models, PARP1 and Erk2 were both activated in neurons exposed to nerve growth factors (NGF, BDNF) that promote memory acquisition and neurite outgrowth [10,15,16]. PARP1 and Erk2 were activated in cortical and hippocampal neurons stimulated by high-frequency electrical stimulation (100 Hz) that induces synaptic long-term potentiation (LTP) [10]. Either MEK or PARP1 inhibitors prevented the expression of immediate-early genes *cfos*, *zif268*, and *arc* in the stimulated neurons [10]. LTP was not generated in the hippocampus of PARP1 KO mice, nor was it observed in the hippocampus of normal mice pretreated with either MEK or PARP1 inhibitors [10].

These results were in accordance with the results obtained by examining the effect of PARP1 activation on “learning” abilities in vivo. The long-term memory of the slug *Aplysia*, as well as that of rodents, was impaired after treatment with PARP1 inhibitors [47,48]. These animals temporarily lost their ability to create a long-term memory of tasks and objects when PARP1 inhibitors were applied 30 min before training, although their previously acquired memories were not impaired [47,48]. These results are in accordance with the identified role of PARP1 polyADP-ribosylation in Erk-induced expression of immediate-early genes that are implicated in synaptic plasticity [10,15,62,63,64]. A PARP1–Erk synergism mediated gene expression in other cells as well [18,42] (Figure 4).

One of the expressed immediate-early genes, *cfos* [67,68,69,70,71], acts after transcription and phosphorylation, as a transcription factor of other genes in a variety of cell types [69,70,71]. Transcription factor cFos, bound to the DNA-binding GATA proteins, acts as a transcription factor in several cell types, including in *ANF* expression in cardiomyocytes, which is crucial for their development and survival [42].

In a variety of healthy and malignant cells, c-Fos bound to Jun combines the transcription factor AP-1, which promotes cell proliferation [68,71,72,73,74]. Growth factors and hormones activate AP-1 via activation of the MAP kinase phosphorylation cascade [33,68]. A variety of other mitogenic signaling pathways in malignant cells converge on the transcription factor AP-1 [33,68,71,72,73]. The dependence of *c-fos* expression on PARP1 activation [10,15,16] may suggest a possible contribution of PARP1 inhibition to the downregulation of uncontrolled Erk-induced gene expression in malignant cells [73,74].

Recently, a long-lasting polyADP-ribosylation of PARP1 has been attributed to the binding of WGR and CAT domains in PARP1 to histone H4, as indicated in a cell-free system [19]. This mechanism may co-exist with Erk-induced PARP1 activation [10,15]. Both mechanisms promote long-lasting PARP1 polyADP-ribosylation in the chromatin. Nevertheless, the activation of PARP1 bound to phosphorylated Erk explains the long-lasting activity of phosphorylated Erk in the chromatin [10,14,15,16,38,40].

The binding of PARP1 to core histones has been implicated in the reassembly of nucleosomes after chromatin relaxation [19], as explained below.

## 5. A Long-Lasting PARP1 PolyADP-Ribosylation Implicated in the Reassembly of Nucleosomes

Recent findings identified a high affinity of polyADP-ribosylated PARP1 to nucleosomes [19,75,76]. While polyADP-ribosylated PARP1 has a low affinity for single-strand DNA, and PARG activity enables PARP1 recurrent binding to the DNA [1,2,3], polyADP-ribosylated PARP1 binds with a high affinity to core histones in the nucleosomes [19,75,76]. The binding of PARP1 via WGR, HD, and CAT domains in PARP1 to histone H4 kept PARP1 polyADP-ribosylated [19,75,76]. This was not the case for un-polyADP-ribosylated PARP1, which has a very low affinity for isolated nucleosomes or histones [19,75,76]. Experiments in cell-free systems showed the ability of polyADP-ribosylated PARP1 to assemble nucleosomes in the absence of DNA damage [19,75,76]. Accordingly, the affinity of polyADP-ribosylated PARP1 for core histones is similar to the measured affinity of other histone chaperones [76]. Binding to polyADP-ribosylated PARP1 induced core histones polyADP-ribosylation as well, but their polyADP-ribosylation was not necessary for their assembly in the nucleosomes [19,76]. In this binding mode of PARP1 to core histones, both the C-terminal of PARP1 and its N-terminal were implicated, while the C-terminal of PARP1 was not implicated in its binding to DNA breaks [19,76]. PolyADP-ribosylated PARP1 exhibited histone chaperone features that were implicated in the reassembly of nucleosomes following stimulation-induced chromatin relaxation and DNA transcription [76]. PARP binding to the nucleosomes restored chromatin compaction [76].

## 6. The Effect of PARP1 PolyADP-Ribosylation on DNA Methylation

A long-lasting PARP1 polyADP-ribosylation is implicated in the interplay between PARP1 polyADP-ribosylation and DNA methylation. This is due to polyADP-ribosylation of the DNA binding protein, CTCF [20,77,78] CTCF binds to DNA CCCTC sites that are prone to DNA methylation [20,77,78]. CTCF co-localizes with PARP1 in its DNA binding sites [20,77,78]. PARP1 binding to CTCF results in a mutual polyADP-ribosylation of PARP1 and CTCF by a yet unclear mechanism [77,78]. PolyADP-ribosylated CTCF is removed from its sites in the DNA [77,78], exposing these DNA sites to methylation [77,78]. DNA methylation causes high-density DNA regions [77], while DNA relaxation and loops formation are observed in CTCF-bound DNA regions [77]. Thus, polyADP-ribosylation causes chromatin remodeling by changing the binding sites of CTCF along the DNA. This mutual CTCF and PARP1 polyADP-ribosylation transiently “locks” or exposes regions in the DNA to either methylation or transcription [20,77,78,79,80]. A massive DNA damage may affect DNA methylation by changing the activity of DNA methyltransferases1 (DMT1). The high affinity of DMT1 for PAR polymers inhibits its activity due to interference of the ADP-ribose polymers with its binding to DNA sites that are prone to methylation [77,78,79]. Therefore, unlike the effect of phosphorylated Erk-induced PARP1 polyADP-ribosylation mediating gene expression, the effect of PARP1 polyADP-ribosylation on DNA methylation may yield different effects on gene expression. Chromatin remodeling by CTCF polyADP-ribosylation and relocation may either promote or suppress the expression of specific genes. In addition, a high polyADP-ribosylation of PARP1 under stress conditions causing massive DNA damage, may cause DNA hypomethylation promoting chromatin de-condensation, which causes genomic instability [77,78,79].

## 7. Conclusions

The well-documented, instantaneous, and short-lasting activation of PARP1 by DNA breaks is only one of the mechanisms inducing PARP1 activation. In the absence of DNA breaks, PARP1 is also activated downstream to networks of signal transduction mechanisms, in response to a variety of extracellular signals. This mechanism results in activation of PARP1 as long as it is bound to phosphorylated Erk translocated to the nucleus. PARP1 bound to phosphorylated Erk has a high affinity for NAD^+^. This is in correlation with its intramolecular modifications involving the movement of HD and catalytic domain, which exposes the NAD^+^-binding site in its catalytic domain. The C-terminal domains were also implicated in the binding of polyADP-ribosylated PARP1 to core histone H4, yielding a long-lasting polyADP-ribosylation of both PARP1 and histone H4. Binding to core histones may implicate polyADP-ribosylated PARP1 in the reassembly of nucleosomes following chromatin relaxation. Another long-lasting polyADP-ribosylation of PARP1 results from the mutual polyADP-ribosylation of PARP1 and the CCCTC DNA binding protein CTCF. The resulting transient relocation of CTCF along the DNA following polyADP-ribosylation affects the chromatin structure and affects gene expression by transiently exposing DNA sites prone to methylation. Finding a mechanism linking extracellular signals activating PARP1 with PARP1 polyADP-ribosylation affecting DNA methylation could implicate PARP1 in a variety of epigenetic mechanisms.
